# Supplementary material for: Clinical usability of 3D gradient-echo-based ultrashort echo time imaging: Is it enough to facilitate diagnostic decision in real-world practice?
Source: PLoS One. 2024 May 9;19(5):e0296696. doi: 10.1371/journal.pone.0296696 (PMC11081383; doi:10.1371/journal.pone.0296696)
Supplement: S1 File — (PDF) [file pone.0296696.s001.pdf]

SUPPLEMENTARY METHODS

MRI acquisition

Briefly, VIBE is a fast T_1-weighted 3D gradient-echo imaging sequence performed during breath-hold. In VIBE, partial Fourier sampling reduces the number of phase-encoding steps, thereby reducing the scan time and allowing for breath-holding data acquisition (Figure 2A). However, sampling that reduces the phase-encoding step decreases the SNR to some extent and through-plane motion in the slab-selective direction can introduce image artifacts. Experimental parameters in VIBE imaging were as follows: TE, 1.3 ms; repetition time (TR), 3.3 ms; flip angle, 9°; matrix size, 600×768; field of view (FOV), 312.5×400×264 mm^3; scan time, 13.37 sec; slice thickness, 3 mm (after interpolation); and in-plane resolution, 0.52×0.52 mm^2.

As shown in Figure 2B, CODE is a 3D gradient-echo-based UTE sequence in which an initial dephasing of the readout gradient is performed while applying a frequency-selective radiofrequency (RF) pulse to minimize TE in the 3D radial acquisition scheme [1]. The excitation bandwidth is typically set equal to the readout bandwidth. Asymmetric echoes are acquired outward from the center of 3D *k*-space (so called “spokes”) along a spiral trajectory on a sphere in the *k*-space. In this study, a SINC-shaped RF pulse tailored by a Hanning window was used for spin excitation. Asymmetric echoes were acquired after the readout gradient reached a plateau. The number of spokes was determined to maintain the *k*-space as uniform as possible after retrospective respiratory gating, using the optimal number of interleaved *k*-space trajectories (or interleaves) [2] as follows: total number of spokes, 130k; number of interleaves, 200; and number of spokes per interleaf, 650. Other experimental parameters in CODE imaging were as follows: TE, 0.18 ms; TR, 3 ms; flip angle, 5°; matrix size, 440×440×440; FOV, 360×360×360 mm^3; scan time, 10 min 40 sec. The nominal

isotropic spatial resolution was 0.82 mm. A spectral preparation pulse was applied for fat suppression every 10 TRs.

Retrospective respiratory gating

Respiratory phase-resolved CODE images were obtained at the end of the expiratory period (or end-expiration) through retrospective respiratory gating. Respiratory signals were extracted from the acquired data, including self-navigating echoes. For self-navigation, dual navigating echoes were acquired periodically in the superior-inferior direction along the spiral *k*-space trajectory and the second navigating echoes, which were closer to steady state, were chosen to minimize the baseline fluctuation of respiratory signals [3]. The sampling interval of self-navigating echoes was set to 49.3 ms, satisfying the Nyquist sampling criterion for respiration rate. Respiratory signals were obtained from the superior-inferior projection profiles by tracking the movement of the diaphragm [3]. Then, CODE images were reconstructed by gating the end-expiratory data, that is, by selecting spokes belonging to the end-expiratory phase of the respiratory signal. The end-expiratory data were gated to obtain more spokes for image reconstruction because expiration is usually longer than inspiration in the respiratory cycle.

REFERENCES

1. Park JY, Moeller S, Goerke U, Auerbach E, Chamberlain R, Ellermann J, et al. Short echo-time 3D radial gradient-echo MRI using concurrent dephasing and excitation. Magn Reson Med. 2012;67(2): 428-436. doi: 10.1002/mrm.23026.

2. Park J, Shin T, Yoon SH, Goo JM, Park JY. A radial sampling strategy for uniform k-space coverage with retrospective respiratory gating in 3D ultrashort-echo-time lung imaging. NMR Biomed. 2016;29(5): 576-587. doi: 10.1002/nbm.3494.

3. Park J, Lee S, Shin T, Oh S-H, Park J-Y. A Robust Self-navigation for Respiratory Gating in 3D Radial Ultrashort Echo-time Lung MRI using Concurrent Dephasing and Excitation. Journal of the Korean Physical Society. 2018;73(1): 138-144.
