# Supplementary material for: Clinical usability of 3D gradient-echo-based ultrashort echo time imaging: Is it enough to facilitate diagnostic decision in real-world practice?
Source: PLoS One. 2024 May 9;19(5):e0296696. doi: 10.1371/journal.pone.0296696 (PMC11081383; doi:10.1371/journal.pone.0296696)
Supplement: S1 Table — (PDF) [file pone.0296696.s002.pdf]

S1 Table. Scanner parameters for VIBE and CODE sequences

	**VIBE**	**CODE**
TR (ms)	3.3	3
TE (ms)	1.3	0.18
Flip angle (°)	9	5
Matrix size	600 × 768	440 × 440 × 440
FOV (mm)	312.5 × 400 × 264	360 × 360 × 360
Respiratory control	Breath hold	Retrospective gating
Slice thickness (mm)	3	0.8182
Resolution (mm)	0.5208 × 0.5208	0.8182 × 0.8182
Mean scan time (sec)	13.37	640

CODE, concurrent dephasing and excitation; FOV, field of view; TE, echo time; TR, repetition time; VIBE, volumetric interpolated breath-hold examination
