# Supplementary material for: Clinical usability of 3D gradient-echo-based ultrashort echo time imaging: Is it enough to facilitate diagnostic decision in real-world practice?
Source: PLoS One. 2024 May 9;19(5):e0296696. doi: 10.1371/journal.pone.0296696 (PMC11081383; doi:10.1371/journal.pone.0296696)
Supplement: S2 Table — (PDF) [file pone.0296696.s003.pdf]

S2 Table. Qualitative grading system for assessment of images obtained using VIBE and CODE sequences

Image quality of central airway and great vessels [1]
(1) Non-diagnostic with strong artifacts
(2) Poor with severe blurring that caused uncertain evaluation
(3) Acceptable with moderate blurring that slightly restricted evaluation
(4) Good with slight blurring that did not restrict evaluation
(5) Excellent without any artifacts
Depictions of intrapulmonary vasculature [2]
(1) No depiction
(2) Depicted at segmental branch level
(3) Depicted at subsegmental branch level
(4) Depicted at sub-subsegmental branch level
(5) Depicted at centrilobular level
Depictions of bronchi [2]
(1) Depicted at main trunk level
(2) Depicted at lobar bronchi level
(3) Depicted at segmental bronchi level
(4) Depicted at subsegmental bronchi level
(5) Depicted at sub-subsegmental bronchi level
Depiction of lesions in lung parenchyma and mediastinum [2]
(1) Absent
(2) Probably absent
(3) Equivocal
(4) Probably present
(5) Present
Extent of emphysema [3]
(1) No emphysema
(2) Mild emphysema (<25%)
(3) Moderate emphysema (25–50%)
(4) Severe emphysema (>50%)

REFERENCES

1. Lee SW, Kim Y, Shim SS, Lee JK, Lee SJ, Ryu YJ, et al. Image quality assessment of ultra low-dose chest CT using sinogram-affirmed iterative reconstruction. Eur Radiol. 2014;24(4): 817-826. doi: 10.1007/s00330-013-3090-9.

2. Ohno Y, Koyama H, Yoshikawa T, Seki S, Takenaka D, Yui M, et al. Pulmonary high-resolution ultrashort TE MR imaging: Comparison with thin-section standard- and low-dose computed tomography for the assessment of pulmonary parenchyma diseases. J

Magn Reson Imaging. 2016;43(2): 512-532. doi: 10.1002/jmri.25008.

3. Benlala I, Berger P, Girodet PO, Dromer C, Macey J, Laurent F, et al. Automated Volumetric Quantification of Emphysema Severity by Using Ultrashort Echo Time MRI: Validation in Participants with Chronic Obstructive Pulmonary Disease. Radiology. 2019;292(1): 216-225. doi: 10.1148/radiol.2019190052.
